# Genetics of Hypertension: From Monogenic Analysis to GETomics

**DOI:** 10.3390/jcdd11050154

**Published:** 2024-05-18

**Authors:** Martina Zappa, Michele Golino, Paolo Verdecchia, Fabio Angeli

**Affiliations:** 1 Department of Medicine and Surgery, University of Insubria, 21100 Varese, Italy; 2 Pauley Heart Center, Virginia Commonwealth University, Richmond, VA 23223, USA; 3 Fondazione Umbra Cuore e Ipertensione-ONLUS, 06100 Perugia, Italy; 4 Division of Cardiology, Hospital S. Maria della Misericordia, 06100 Perugia, Italy; 5 Department of Medicine and Technological Innovation (DiMIT), University of Insubria, 21100 Varese, Italy; 6 Department of Medicine and Cardiopulmonary Rehabilitation, Maugeri Care and Research Institutes, IRCCS, 21049 Tradate, Italy

**Keywords:** arterial hypertension, genetics, gene–environment interactions

## Abstract

Arterial hypertension is the most frequent cardiovascular risk factor all over the world, and it is one of the leading drivers of the risk of cardiovascular events and death. It is a complex trait influenced by heritable and environmental factors. To date, the World Health Organization estimates that 1.28 billion adults aged 30–79 years worldwide have arterial hypertension (defined by European guidelines as office systolic blood pressure ≥ 140 mmHg or office diastolic blood pressure ≥ 90 mmHg), and 7.1 million die from this disease. The molecular genetic basis of primary arterial hypertension is the subject of intense research and has recently yielded remarkable progress. In this review, we will discuss the genetics of arterial hypertension. Recent studies have identified over 900 independent loci associated with blood pressure regulation across the genome. Comprehending these mechanisms not only could shed light on the pathogenesis of the disease but also hold the potential for assessing the risk of developing arterial hypertension in the future. In addition, these findings may pave the way for novel drug development and personalized therapeutic strategies.

## 1. Hypertension: Definition, Global Impact, and Genetic Pathogenesis

Hypertension, or elevated blood pressure (BP), occurs when either systolic blood pressure (SBP), diastolic blood pressure (DBP), or both are above recognized normal levels. This condition is widespread across developed and developing countries and is influenced by age, sex, genetics, and demographic factors.

Over time, the adjustments of the diagnostic criteria for hypertension reflect findings from studies that have shown benefits in reducing mortality and several cardiovascular events such as chest pain and heart failure. According to the 2018 European Society of Cardiology/European Society of Hypertension guidelines, arterial hypertension is defined as a SBP of 140 mmHg or higher and/or a DBP of 90 mmHg or higher [1]. On the other hand, the 2017 guidelines from the American College of Cardiology-American Heart Association (ACC-AHA) set the threshold at a SBP of 130 mm Hg or higher or a DBP of 80 mmHg or higher [2].

In the United States, hypertension is more prevalent among men, elderly, African Americans, and those with obesity, chronic kidney disease, and diabetes. Additionally, its incidence is notably higher in rural areas [3,4,5]. Its prevalence is comparable globally, although it varies between countries [6,7]. In 1990, approximately 32% of adults aged 30–79 years had hypertension [4].

Epidemiological studies have documented a prevalence of hypertension of 55% among participants in Europe, higher than in other regions [8].

In 2019, about 1.3 billion adults worldwide were affected by hypertension, with 82% living in low- and middle-income countries [4]. Interestingly, the diagnostic criteria for hypertension vary across countries [1]. However, by applying the 2017 ACC/AHA hypertension guidelines globally, we could potentially identify upwards of 1.8 billion individuals with hypertension.

According to a recent study, the number of people aged 30–79 years with hypertension has changed dramatically from 1990 to 2019: from 331 million women and 317 million men in 1990 to 626 million women and 652 million men in 2019, despite stable global age-standardized prevalence [4].

Furthermore, uncontrolled hypertension is linked to 7.6–9 million premature deaths per year, with a SBP ≥ 140 mmHg contributing to about 70% of the global mortality and morbidity burden [9,10].

The pathogenesis of primary hypertension is still poorly understood, with few innovative clinical studies regarding its treatment [11]. To date, many studies have been published in the literature on genes or chromosome loci linked to hypertension, but there is a need for further studies to explore this field [12,13,14,15,16,17]. This difference is probably partly due to the different sample size or different population origin. In addition, as reported by Surina Singh and colleague, most studies have been conducted on European populations and very few on African populations, where African populations have high genetic diversity, allele frequency differences, and low linkage disequilibrium when compared to other populations [18].

Genetic factors are estimated to contribute about 20–55% to hypertension [19]. Identifying specific genes for hypertension is challenging due to population diversity, genetic heterogeneity, and other confounding factors. In this context, it is now well recognized that hypertension results from a complex interplay of several genetic and environmental factors, all affecting cardiovascular structure and function.

The main aim of this review is to summarize accrued data on the role of genetic alterations in the pathogenesis of hypertension as well as explore the potential of pharmacological intervention in its management.

## 2. Familial Aggregation and Heritability

Family aggregation, also known as familial aggregation, refers to the clustering of specific traits, behaviors, or disorders within families. In the literature, this concept often refers to the degree to which children exhibit the same behaviors as their parents [20,21].

With regard to hypertension, the initial studies were conducted in the 1960s/1970s and showed that the family tendency towards high and low pressure was established early in life [22]. However, whether this aggregation within families was due to genetic factors or the shared family environment was unclear. This knowledge gap led to more focused research over the years, focusing on the twin population [23]. Interestingly, most of these studies found strong evidence of genetic influence on BP, with little impact from the shared family environment. It is estimated that about 50% of the variance in BP is attributed to genetic factors [23], while environmental factors contribute about 10–20%.

Although family clustering is influenced by both genetic and environmental factors, in reality, these two components exhibit close connections. For instance, obesity, the most important risk factor for hypertension, shows a strong familial trend. Other apparently ‘environmental’ risk factors, like sodium intake [20,24] and parental socio-economic status [25], also have genetic components. This could also explain why twin studies predominantly identified genetic influences but not shared family environment factors in childhood BP [23].

Furthermore, a 2008 research that followed 1160 male former medical students for 54 years found that participants with both parents having high blood pressure had a 2.4-fold increased risk of developing hypertension [26]. In a similar vein, Stamler et al. discovered in a sizable cross-sectional research that a parent’s history of hypertension increased the likelihood of having the condition [27].

Cross-sectional studies can have certain drawbacks, too, such as recollection bias. Young people’s prospective studies have made an effort to reduce this bias by evaluating participants’ family histories prior to the onset of hypertension. After adjusting for age and length of follow-up, the first published prospective study found that males with a history of hypertension in their parents had a 1.7-fold greater incidence of hypertension.

Contrary to the BP correlation findings, a case-control study using health plan data reported that a parental history of hypertension was a significant predictor only in women, with a maternal history of hypertension being a stronger predictor [28].

## 3. Monogenic Forms of Hypertension

Monogenic disorders result from the transmission of single gene mutations. Alternatively, a monogenic disorder can occur due to a de novo mutation in either the paternal or maternal germ line.

Monogenic hypertension (MH) is a rare form of arterial hypertension (AH) caused by a single gene mutation, following the principles of Mendelian inheritance.

MH can arise from germline or somatic mutations with dominant or recessive inheritance. These mutations typically affect around 35 genes encoding enzymes or receptors, often leading to increased renal sodium reabsorption, excessive aldosterone production, and adrenal steroid metabolism dysregulation. Consequently, these changes can lead to an expansion of plasma volume and a significant rise in BP, typically by 20–50 mmHg [29,30].

Genetic diagnosis of these monogenic disorders is important, as the therapy is specific to the underlying genetic abnormality.

To date, there are different genetic syndromes associated with MH. It is important to note that some may present with only a mild increase in BP, with or without transient or borderline biochemical disorders. The principal monogenic diseases are summarized in Table 1 and listed below:

- Liddle’s syndrome (LS), one of the most common MH forms, is a rare and autosomal dominant disorder (AD) caused by mutations in sodium channel epithelial 1 subunit alpha (*SCNN1A*), sodium channel epithelial 1 subunit beta (*SCNN1B*), and sodium channel epithelial 1 subunit gamma (*SCNN1G*) genes. These genes encode the α, β, and γ subunits of the epithelial sodium channel (*ENaC*) [31,32,33]. LS is often associated with a family history of sudden death and early hypertension. Characteristic features include hypokalemia, metabolic alkalosis, suppressed plasma renin activity, and low plasma aldosterone [31,34]. To date, 31 mutations leading to LS have been identified, with different symptoms among patients [31]. Worldwide, fewer than 30 families (or isolated cases) have been described [35].

Moreover, several mutations affecting *ENaC* activity can lead to a Liddle-like phenotype, distinct from LS.

- Gordon’s syndrome, or pseudohypoaldosteronism type 2 (PHA II), results from mutations in four genes regulating Na-K-Cl cotransporter activity in the distal convoluted tubules of the kidney (NCC). It is a rare and inherited form of low-renin hypertension associated with hyperkalemia and metabolic acidosis [36,37], first described by Cheek and Perry in 1958 [38]. The implicated genes are cullin 3 (*CUL3*), kelch-like family member 3 (*KLHL3*), WNK lysine-deficient protein kinase 1 (*WNK1*), and WNK lysine-deficient protein kinase 4 (*WNK4*) [39,40]. Mainly, a loss-of-function mutation in the *WNK4* gene located on chromosome 17 (17q21.2) directly inhibits the NCC activity by reducing its expression on the extracellular membrane [40]. Hyperkalemia is a feature of Gordon’s syndrome, but due to the heterogeneity of the mutations, different patients exhibit normal potassium levels and different levels of severity of hypertension. The *CUL3* mutation (autosomal recessive) leads to a more severe and earlier onset of symptoms, while the *WNK1* mutation (autosomal dominant) is associated with a milder phenotype [41]. In more severe cases, patients may present with short stature and intellectual disability.
- Glucocorticoid remediable aldosteronism (GRA) or familial hyperaldosteronism I (FH I) is caused by a chimeric gene in which the ACTH-responsive 5′-promoter of the 11Beta-hydroxylase gene is fused to coding sequences of the aldosterone synthase gene, resulting in a hybrid protein that stimulates aldosterone production independently of renin [42]. This disorder is characterized by moderate to severe early-onset AH, low plasma renin, mild hypokalemia despite high aldosterone levels, and metabolic alkalosis [30,43]. GRA often presents as asymptomatic severe hypertension in infancy or early adulthood and carries a high risk of hemorrhagic stroke due to ruptured intracranial aneurysms [44].
- Apparent mineralocorticoid excess (AME) is a rare autosomal recessive disorder caused by a deficiency in the 11β-hydroxysteroid dehydrogenase type 2 (*11βHSD2*), which physiologically converts cortisol to cortisone, thus protecting mineralocorticoid receptor (MR) activation by cortisol [45,46,47,48]. Consequently, the serum cortisol half-life (T½) is prolonged, ACTH is suppressed, and serum cortisol concentration is normal. Activation of the MR induces AH, hypokalemia, and metabolic alkalosis.
  The clinical symptoms of AME were first reported in 1974 by Edmond A. Werder in a 3-year-old girl with low birth weight, delayed growth, polydipsia, polyuria, and hypertension. Only in 1995 the first *HSD11B2* mutation was identified in a consanguineous Iranian family, revealing the genetic substratum of the disease [45]. The clinical manifestation of AME is heterogeneous, with a solid phenotype–genotype correlation. Nephrocalcinosis due to hypercalciuria is observed in 50–75% of the cases, and some experience kidney cysts due to chronic hypokalemia [49].
- Geller syndrome is a rare autosomal dominant disorder resulting from a gain-of-function mutation on chromosome 4q31, affecting the mineralocorticoid receptor and leading to increased affinity of certain steroid hormones such as progesterone [50,51]. The mutation, S810L, was first described by Geller et al. and caused early-onset hypertension exacerbated in pregnancy [52]. This mutation results in the gain of a van der Waals interaction between helix-5 and helix-3 [53]. Clinically, this presents as hypertension and hypokalemia during high progesterone states, such as pregnancy. Thus, every pregnancy in a patient with Geller syndrome should be closely monitored [54].
- Congenital adrenal hyperplasia (CAH) describes a group of hereditary disorders affecting the adrenal glands. The two main types of CAH are classic and non-classic [55], with the first diagnosed at birth and the second typically diagnosed during adolescence. Two forms of CAH, 11β-hydroxylase deficiency (11β-OHD, type IV) [56] and 17α-hydroxylase deficiency (17α-OHD, type V), are associated with early-onset hypertension and hypokalemia [57]. In both subtypes, hypertension results from the overproduction of intermediate products with affinity to MR activation. Type IV accounts for 0.2–8% of all CAH cases [58] and is marked by rapid somatic growth and skeletal maturation due to hyperandrogenemia, leading to virilization in girls and premature puberty in boys [59]. In two-thirds of patients, AH appears during the first decade of life. However, the onset in the newborn period has been described. Adrenal crises in patients with classic CAH have been described. This is a life-threatening medical emergency that requires immediate treatment.

## 4. Polygenic Hypertension

Many independently functioning or interacting polymorphic genes work together to generate polygenic disorders; each gene’s individual impact may be negligible or even invisible. The presence of clinically diverse types of the disease and the effectiveness of treatment can be predicted by the carriage of specific gene combinations.

Hypertension is a complex polygenic disease (Table 2), and identifying the genes involved in its etiology can lead to a greater understanding of the primary pathogenic mechanisms of hypertension, target organ complications, and interactions with environmental factors. Large-scale association studies that examine many gene polymorphisms simultaneously are crucial for predicting genetic risks.

In a complex multifactorial pathology, single gene variants have limited informative value in assessing BP risk due to their minor individual impact. The field has evolved significantly since the first genome-wide association studies (GWAS) for hypertension were conducted in 2007, 4 years after the human genome was sequenced, but yielded disappointing results [60,61]. In particular, in the first one, the researcher observed association at many previously identified loci and found that some loci confer risk for more than one of the diseases studied; in the second one, instead, the genome-wide associations for blood pressure and arterial stiffness were evaluated. The results highlighted that there are no associations that attained genome-wide significance. They found only weak association of SNPs in the renin-angiotensin-aldosterone pathway with BP or arterial stiffness.

One year later, in 2008, the first locus (ATP2B1 = ATPase plasma membrane Ca^2+^ transporting 1) significantly associated with BP was identified in the GWAS of 1484 Japanese individuals [62].

The *ATP2B1* gene encodes for the plasma membrane calcium ATPase isoform 1, which plays a key role in blood pressure regulation through altered calcium handling and vasoconstriction of vascular smooth muscle cells. In particular, single nucleotide polymorphism (SNP) rs2681472 in the *ATP2B1* gene was identified to be associated with blood pressure or hypertension in different populations.

However, more recent consortia of GWAS have identified several new loci for BP by expanding the number of studies included in meta-analyses, leading to a significantly greater number of genetic determinants associated with various BP-related traits [17,63].

In particular, Evangelou and colleagues identified 535 new BP loci that offer further information on BP regulation and highlight a shared scaffold between BP and lifestyle [17].

Additionally, a study conducted on a cohort of 1940 unrelated Japanese (including 1067 hypertensive patients and 873 controls) evaluated 33 single nucleotide polymorphisms in 27 candidate genes [64]. The results showed that two polymorphisms (825C→T in the G protein β3 subunit gene and 190G→A in the CC chemokine receptor 2 gene) were associated with male hypertension.

Moreover, another polymorphism (−238G→A in the tumor necrosis factor α gene) was significantly associated with an increased BP in women. These findings suggest that the tumor necrosis factor α gene is a candidate locus for susceptibility to hypertension; in particular, the A allele of the −238G→A SNP of this gene protects against the development of hypertension [63]

However, despite identifying over 1000 loci for BP, these explain only 6% of heritability based on SNPs [17,65]. In addition, many of these loci are located in non-coding regions, making it difficult to understand their functions [66].

To overcome this problem, transcriptome-wide association studies (TWAS) have been introduced, combining individual-level genotype data or GWAS summary data with quantitative expression trait loci (eQTLs) to assess gene expression level associations with diseases [67]. This gene-based association approach with tissue specificity has a lower multiple-testing burden [68] and has identified three key genes in essential hypertension: potassium two-pore domain channel subfamily K member 3 (*KCNK3*), glutamyl aminopeptidase (*ENPEP*), and ubiquitin-specific peptidase 38 (*USP38*) [67]. *KCNK3* encodes for potassium two-pore domain channel subfamily K member 3, an outwardly rectifying channel protein sensitive to changes in extracellular pH and inhibited by extracellular acidification. KCNK3 was also identified to be associated with blood pressure in a genome-wide association study [69]. The *ENPEP* gene is responsible for encoding glutamyl aminopeptidase, which is a type II integral membrane protein featuring an extracellular zinc-binding domain. This particular protein has the ability to increase blood pressure by cleaving the N-terminal aspartate from angiotensin II. Additionally, it plays a role in regulating the formation of blood vessels and promoting tumorigenesis in certain tissues [70]. The gene ENPEP had been found to be associated with hypertension in a meta-analysis [71]. However, these three genes were not reported in one of the largest GWAS studies on hypertension by Evangelou et al. [17].

Notably, until the middle of 2017, GWAS have identified and replicated genetic variants of modest or weak effect on BP over 200 loci [72]. In 2017, a study group incorporated GWAS data from 330,956 individuals and revealed 107 significant loci, of which 24 were linked with systolic blood pressure (SBP), 41 with diastolic blood pressure (DBP), and 42 with PP [73]. However, the biggest genetic association research of pulse, diastolic, and systolic blood pressure features in over a million adults of European ancestry was published in 2018. They discovered 535 new blood pressure loci, which demonstrate common genetic architecture between blood pressure and lifestyle stressors in addition to providing fresh biological insights into blood pressure control [17].

Regarding the Asiatic population, the Asian Genetic Epidemiology Network (AGEN) group published the first extensive meta-analysis of GWAS on BP characteristics among East Asians [15]. Following de novo genotyping in two replication stages involving 10,518 and 20,247 East Asian samples, Lu and colleague found six novel loci (*ST7L-CAPZA1*, *FIGN-GRB14*, *ENPEP*, *NPR3*, *TBX3*, and *ALDH2*) [74].

Compared to European or Asian populations, fewer loci in African populations reach GWAS significance. The largest GWAS conducted in a population of African descent examined 21 GWAS with 31,968 African ancestors, and it confirmed its findings with an additional 54,395 participants from multi-ethnic studies [75]. For either combined characteristics, hypertension, DBP, or SBP, the authors identified 9 loci containing 11 independent variations.

## 5. Gene–Environment Interactions

Over the years, doctors and scientists have tried to figure out how diseases start. Although certain diseases can be attributed to a singular factor, the etiology of intricate diseases presents a more stimulating challenge, primarily due to the interconnected nature of numerous contributing factors. One factor that contributes to the risk of disease is an individual’s genetics. Certain individuals inherit specific genetic variants that either directly initiate disease pathogenesis or work in concert with other factors and/or other genetic variants to augment the risk of disease.

It has also been demonstrated that environmental exposures—here defined as chemicals, infections, and other external factors—often have a role in the development of disease. Not every person exposed to a particular environmental factor will acquire an illness, even if epidemiological studies might show associations between exposure to environmental variables and disease etiology. Similarly, not everyone who inherits a specific genetic mutation goes on to become sick. It is clear that combinations of antagonistic or synergistic variables influence illness risk for the great majority of diseases.

A gene–environment interaction occurs when there is an interplay between genotype, phenotype, and the environmental context. The word ‘environment’ defines almost all non-genetic factors to which cells or organisms are exposed.

**Figure 1:**
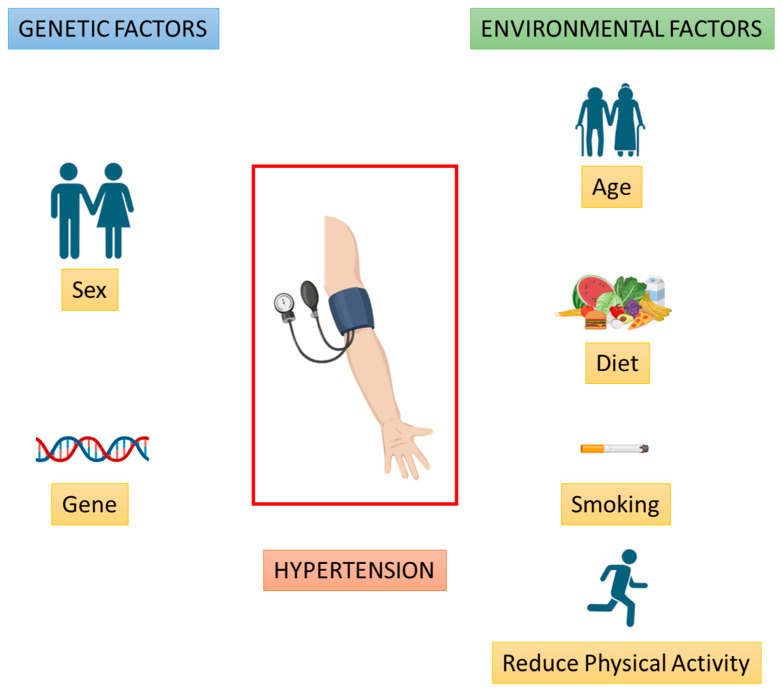
Overview of the main genetic and environmental factors involved in hypertension (Created with BioRender.com).

There is growing evidence that complex interactions between several genes and several environmental factors (G × E) play an important role in determining the risk of various common diseases. This concept particularly applies to hypertension, where the genetic predisposition can be significantly influenced by environmental factors such as stress, diet, lifestyle [76,77], smoking [78], and obesity [65,79] (Figure 1).

The GWAS for HTN-related phenotypes has identified significant interactions between genetics and environmental factors such as alcohol consumption [80], body mass index (BMI) [81], smoking [82,83], education levels [84], and sodium intake [85]. All these factors are particularly notable because they can be modified through lifestyle changes [86].

In addition, recent epidemiological and experimental studies suggest that even paternal environmental factors, before conception and during sperm development, might be linked to the development of hypertension in later life [87].

A study involving 1575 Chinese individuals identified a significant interaction between alcohol consumption and hypertension risk linked to a polymorphism (−344TC) in the cytochrome P450 family 11 subfamily B member 2 (*CYP11B2*) gene. This gene encodes a member of the cytochrome P450 superfamily. There is a correlation between CYP11B2 polymorphism and a higher risk of hypertension. It was previously discovered that the CYP11B2 gene had several frequent polymorphisms [88,89]. Moreover, it was previously discovered that the CYP11B2 gene had several frequent polymorphisms. Hyperaldosteronism, glucose intolerance and plasma glucose levels [90], type II diabetes mellitus (DM) [91], left ventricular mass and size [92], and arterial stiffness and myocardial infarction [93] are all associated with these polymorphisms.

In particular, in subjects who consumed 200 g/d of alcohol, the risk of hypertension was significantly higher in carriers of the TT alleles compared to the CC genotype [94].

Another study investigated the influence of occupation and community on BP regulation. Menni et al. conducted a study involving 924 European participants and found that the perceived work control had a significant impact on the relationship between a specific polymorphism (rs11210278) of the endothelin 2 (*EDN2*) gene, responsible for encoding endothelin-2, and ambulatory systolic blood pressure (SBP) [76]. Additionally, a similar interaction was observed with the endothelin 1 (EDN1) gene, where individuals who were homozygous carriers of the rs5369-G allele and reported experiencing work-related stress had notably higher SBP compared to heterozygotes [95].

Regarding smoking habits, a study conducted in the rural Chinese population revealed a minimal impact of cigarette smoking on ACE gene expression, specifically in relation to essential hypertension [96]. Conversely, in an eastern Han Chinese population, the interaction between the rs1126742 SNP and smoking was found to be associated with a heightened risk of essential hypertension [97]. Furthermore, a case-control study demonstrated a correlation between potassium inwardly rectifying channel subfamily J member 11 (*KCNJ11*) gene polymorphisms and the blood pressure response to the antihypertensive drug irbesartan in Chinese hypertensive patients who do not smoke [96,97,98].

To sum up, gene–environment interactions can also be associated with hypertension, but adopting a lifestyle that involves weight reduction (in the case of obese patients), a healthy diet, the reduction of sodium in the diet, increased physical activity, quitting smoking, and moderate alcohol consumption can significantly influence the onset and development of the disease.

## 6. Road to GETomics

In 2022, Prof Alvar Agustí and colleagues introduced the term “GETomics” to illustrate that human health and disease are actually the result of many interactions between genes (G) and environment (E) that occur throughout an individual’s lifetime (T). The researchers pointed out that each GxE interaction is influenced by the age of the individual at which that interaction occurs and by previous GxE interactions. The authors proposed this integrative approach, using multiple omics platforms to improve understanding of chronic obstructive pulmonary disease pathogenesis in the context of complex interactions between genes (G) and environment (E) over time (T) [99]. A novel dynamic and comprehensive knowledge of the pathophysiology of human conditions in general is offered by the notion of GETomics. Therefore, it refers to the processes that control the treatable characteristics that are present in every specific patient and underlie the clinical presentation.

The GETomics concept holds that gene–environment interactions begin from conception and persist through life to death. These interactions trigger biological reactions, such as innate or acquired immunological responses, which in turn affect organ structure (development, maintenance and repair, aging, and so on) and function. As a result, two more crucial elements need to be taken into account: biological memory and age.

This approach has also been proposed for other medical conditions [100,101] and could be relevant for several chronic human diseases like hypertension.

Blood pressure is the product of cardiac output and peripheral vascular resistance, which are regulated by different factors. Therefore, blood pressure is a multifactorial trait with complex molecular mechanisms influencing the final phenotype.

As said before through investigations of gene–environment (GxE) interactions, the role of genetics in the pathophysiology of hypertension may also be explained. The premise of GxE research is that people could be more susceptible to the detrimental impacts of environmental adversity or, conversely, more receptive to favourable environmental experiences. Additional blood pressure-associated loci that can only be found by modifying or interacting with environmental exposure can also be found using GxE investigations.

However, traditionally, increased BP has been viewed as a natural aspect of aging, often leading to hypertension in the elderly. This understanding has evolved significantly. In this context, epigenetic regulation plays an important role, which can alter gene expression without changing the nucleotide base sequence of the gene and can result from environment–gene interactions.

Conventional talks on hypertension have mostly concentrated on the dangers of cardiovascular disease and its side effects. Nonetheless, a variety of unintended consequences, such as increased susceptibility to dementia, physical impairment, and falls/fractures, are drawing more and more attention in the research on hypertension. Inflammation, oxidative stress, and endothelial dysfunction are among the major pathways shared by biologic aging and the development of hypertension. These mechanisms also seem to play important roles in the development of the cardiovascular and incidental hazards associated with late-life hypertension.

However, the conceptual framework for this comprehensive strategy, known as GETomics and focused on the variety of interactions that occur throughout an individual’s lifespan between genes and environment, has already been established and just needs to be applied to clinical practice.

For all these reasons, probably also in the context of hypertension we will have to move towards the evolution of the GxE interaction concept by also considering the time factor as a pivotal concept within this link.

## 7. Future Directions and Research Opportunities

Although cardiovascular diseases remain a leading cause of death worldwide, the development of new drugs for high BP has slowed considerably. From 2000 to 2017, only six new molecules were approved by the Food and Drug Administration (FDA) for treating hypertension. This highlights a significant gap in effectively managing hypertension and its associated cardiovascular risks, underscoring the need for ongoing clinical and research efforts to find more effective treatments.

To date, two gene therapy approaches for hypertension are in the experimental phase. One is the delivery of antisense oligonucleotides directed at gene targets relevant to current drug therapy. The second involves using viral vectors to deliver multiple copies of ‘vasodilator genes’ or to inhibit ‘vasoconstrictor genes’.

In 2023, a phase 1 study was published to evaluate an RNA interference therapeutic agent for hypertension. The primary outcome of this study was to assess the safety, tolerability, pharmacokinetics, and pharmacodynamics effects of subcutaneous ALN-AGT01 (zilebesiran) in participants with hypertension. The secondary endpoints were the change from baseline in the serum angiotensinogen level. The study involved 107 patients with hypertension, and during the study, there were no deaths or unplanned hospitalizations, and no patients received interventions for hypotension, hyperkalemia, or worsening of renal function. The research demonstrated that a single dose of zilebesiran could decrease serum angiotensinogen levels and 24 h ambulatory BP. This effect represents a significant advancement in hypertension treatment, especially considering that no new drug classes have been approved for hypertension in recent years. This new approach using siRNA leads to a substantial reduction in BP throughout the day. Moreover, the effect of the therapy lasts for about six months after a single injection, avoiding the difficulties of medication adherence encountered with common hypertension drugs.

The ongoing study, KARDIA-2 (NCT05103332), aims to evaluate the effect of zilebesiran on SBP and DBP and characterize its pharmacodynamic effects and safety as an add-on therapy.

## 8. Conclusions

Hypertension is a multifactorial disease and one of the most common cardiovascular risks, affecting about 30–45% of the general population. Moreover, its prevalence fell from 52.8% between 2009 and 2012 to 48.2% from 2017 to 2020 (P trend, 0.029) according to the National Health and Nutrition Examination Survey Report (NHANES) [102].

Investigating the underlying genetics of hypertension and its association with environmental factors is extremely important not only from a scientific but also from a therapeutic point of view, as modulation of gene expression can be used as a therapeutic target.

Indeed, the drugs aimed at controlling hypertension are aimed at relatively few targets. This limitation indicates that antihypertensive therapy is still an open field for improvement and regulation of specific genes involved in hypertension may be an area for further development.

From a therapeutic point of view, although there are currently multiple effective and well-tolerated drugs for the treatment of hypertension, further studies are needed to optimize their use, whether individually or in combination. Unfortunately, research into new antihypertensive drugs has notably slowed down in recent years. In agreement with experts like Bhudia [103] and Verdecchia [11], the future of the management of hypertensive patients remains uncertain. However, significant progress will likely occur in the coming years due partly to the introduction of new biological drugs on the market. These advancements will potentially revolutionize our approach to hypertension and greatly enhance the health and well-being of those affected.

## Figures and Tables

**Table 1:** Characteristics of monogenic forms of hypertension.

Disorder	Age of Onset	Pattern of Inheritance	Aldosterone Level	Serum Potassium Level
Liddle syndrome	Third decade	Autosomal dominant	Low	Low to Normal
Gordon’s syndrome	Second or third decade	Autosomal dominant	Low	High
FH-I (GRA)	Second or third decade	Autosomal dominant	High	Decreased in 50% of cases
AME	Childhood	Autosomal recessive	Low	Low to Normal
Geller syndrome	Unknown	Autosomal dominant	Low	Low to Normal
CAH	Childhood	Autosomal recessive	Low	Low to Normal

Abbreviations: GRA: glucocorticoid remediable aldosteronism; AME: apparent mineralocorticoid excess; CAH: congenital adrenal hyperplasia.

**Table 2:** The principal discovered genes and their location, polymorphism, and genotype that play a role in arterial hypertension (HTN).

Gene	Location	Polymorphism	Genotype	Effect
*11B-HSD1*	1q32.2	rs45487298	A/A	Increase
*5-HTT*	17q11.2	-	L/S	Increase
*5-HTTLPR*	17q11.2	rs25531	S/S	Increase
			S/S	Increase
*ACE*	17q23.3	-	D/D	Increase
			I/D	Increase
*ADD1*	4p16.3	Gly460Trp	T/T	Increase
			G/T	Increase
*AGT*	1q42.2	RS699 M235T	GIG	Increase
			T/T	Increase
			M/T	Increase
			G/G	Increase
*AGTR1*	3q24	A1166C	AC	Increase
		rs4524238	G/G	Increase
*APOE*	19q13.32	rs7412	T/T	Increase
*COX-2*	1q25.2–q25.3.9	rs5275	T/C	Increase
		rs20417	G/C	Increase
*DRD2*	11q23.2	−141c Ins/Del	D/D	Increase
*EDN1*	6p24.1	1rs5370 rs397751713 LYS198ASN	-	Increase
			A/A	Increase
			Asn/Asn	Increase
			Lys/Asn	Increase
*ERBB3*	12q13.2	rs705708	A/A	Decrease
*HIF1α*	14q23.2	rs12434438	A/A	Increase
*IRS-1*	2q36.3	rs2943640	C/C	Increase
			C/A	Increase
*ITGA2*	5q11.2	rs1126643	T/T	Increase
			C/T	Increase
*MMP-9*	20q13.12	rs11697325	A/A	Increase
*MTHFR*	1p36.22	C677	T/T	Increase
		C677T	T/T	Increase
*NOS2*	17q11.2	rs2779249	C/A	Increase
		rs2297518	G/A	Increase
		rs1800482	C/C	Increase
		rs3730017	T/T	Decrease
*RASA3*	13q34	rs9525228	E/A	Increase
*RNF213*	17q25.3	rs112735431	G/A	Increase
*SCNN1A*	12p13.31	rs11064153	T/T	Increase
			C/C	Decrease
*TBX2*	17q23.2	rs8068318	T/C	Increase
*TNF*	6p21.33	G308A	G/A	Increase
*TNFR2*	1p36.22	rs1061624	A/A	Increase
*VDR*	12q13.11	FokI	f/f	Decrease
*WNK4*	17q21.2	rs137853092	C/G	Increased

Abbreviations: *11B-HSD1*, 11 beta hydroxysteroid dehydrogenase type 1; *5-HTT*, serotonin transporter; *5-HTTLPR*, serotonin-transporter-linked promoter region; *ACE*, angiotensin-I converting enzyme; *ADD1*, alpha-adducin 1; *AGT*, angiotensinogen; *AGTR1*, angiotensinogen II, type 1 receptor; *APOE*, apo lipoprotein EF; *COX2*, cytochrome c oxidase subunit 2; *DRD2*, D2-dopaminergic receptor; *EDN1*, endothelin1; *ERBB3*, erb-b2, receptor tyrosine kinase 3; *HIF1α*, hypoxia-inducible factor 1; *IRS-1*, insulin receptor substrate-1; *ITGA2*, integrin subunit alpha 2; *MMP-9*, matrix metalloproteinase-9; *MTHER*, methylenetetrahydrofolate reductase; *NOS2*, nitric oxide synthase 2; *RASA3*, Ras GTPase, activating protein 3; *RNF213*, ring finger protein 213; *SCNN1A*, sodium channel epithelial 1 subunit alpha; *TBX2*, T-box transcription factor 2; *TNF*, tumor necrosis factor; *TNFR2*, tumor necrosis factor receptor 2; *VDR*, vitamin D receptor; *WNK4*, lysine-deficient protein kinase 4.

## Data Availability

Not applicable.
